# Antigen-presenting fibroblasts: emerging players in immune modulation and therapeutic targets

**DOI:** 10.7150/thno.104900

**Published:** 2025-02-18

**Authors:** Xiaoyun Chen, Fangqi Chen, Sujie Jia, Qianjin Lu, Ming Zhao

**Affiliations:** 1 Department of Dermatology, Hunan Key Laboratory of Medical Epigenomics, the Second Xiangya Hospital, Central South University, Changsha, 410011, China.; 2 Institute of Dermatology, Chinese Academy of Medical Sciences and Peking Union Medical College, Nanjing, 210042, China.; 3 Key Laboratory of Basic and Translational Research on Immune-Mediated Skin Diseases, Chinese Academy of Medical Sciences, Nanjing, China.

**Keywords:** antigen presenting fibroblast, MHC-II, T cell, immune regulation, therapeutic potential

## Abstract

Antigen-presenting fibroblasts are a newly recognized subset that challenges the traditional view of these cells as mere structural components. Under pathological or environmental stimuli, fibroblasts acquire antigen-presenting capabilities through the expression of MHC-II molecules and co-stimulatory factors, enabling them to interact with T cells and modulate immune responses. These specialized fibroblasts have been identified across various tissues and diseases, where they play context-dependent roles, either amplifying immune dysregulation or contributing to immune homeostasis. This review synthesizes recent advances in understanding the origins, activation, and functions of antigen-presenting fibroblasts. It highlights their role in promoting pathogenic immune responses and offering therapeutic opportunities through targeted modulation. Advancing our understanding of antigen-presenting fibroblasts holds great promise for developing innovative approaches to immune modulation and therapy across a range of diseases.

## 1. Introduction

Antigen presentation is a cornerstone of the immune system, driving adaptive immune responses through the activation of T cells 1, 2. Classical antigen-presenting cells (APCs), such as dendritic cells, macrophages, and B cells, are central to this process 3. Dendritic cells are the most potent initiators of primary T cell responses, while macrophages and B cells bridge innate and adaptive immunity by facilitating inflammation and humoral responses, respectively 4-7. Professional APCs have long been seen as the primary mediators of antigen-driven immune activation.

Recent discoveries, however, challenge this established paradigm by revealing the antigen-presenting potential of fibroblasts 8. Traditionally considered structural cells that maintain tissue integrity and regulate extracellular matrix composition, fibroblasts have now been shown to acquire antigen-presenting capabilities under pathological conditions, such as chronic inflammation, autoimmune diseases, and tumors 9. This redefines fibroblasts as active players in immune regulation rather than passive structural components.

This review aims to consolidate recent advances in antigen-presenting fibroblasts research, with a focus on their tissue-specific distribution, mechanisms of activation, and functional roles in immune modulation. By offering a comprehensive overview, this review positions antigen-presenting fibroblasts as a promising focus for future research and therapeutic innovation 10, 11.

## 2. Discovery and distribution of antigen-presenting fibroblasts

In the early twentieth century, HLA-DR-positive fibroblasts were found to present tetanus toxoid (TT) to autologous TT-specific monoclonal helper T cells in an MHC-restricted manner, and hypothesis the antigen presentation function of human dermal fibroblasts 12. However, this hypothesis remained largely unexplored due to the technological limitations of the time. The advent of advanced techniques, such as single-cell RNA sequencing and high-throughput molecular profiling, has dramatically transformed our understanding of fibroblast heterogeneity and their roles in immune regulation 13, 14. This heterogeneity allows for the classification of fibroblasts into distinct subtypes, each with unique gene expression profiles that reflect their contributions to tissue homeostasis and diseases 15, 16. Antigen-presenting fibroblasts are characterized by their ability to express MHC-II molecules (HLA-DR, HLA-DP, HLA-DQ) and CD74, which are traditionally associated with professional APCs 17. Notably, these fibroblasts also upregulate co-stimulatory molecules (CD40, CD80, CD86) under certain pathological conditions, which are critical for effective T cell interaction and immune activation.

**Figure 1:**
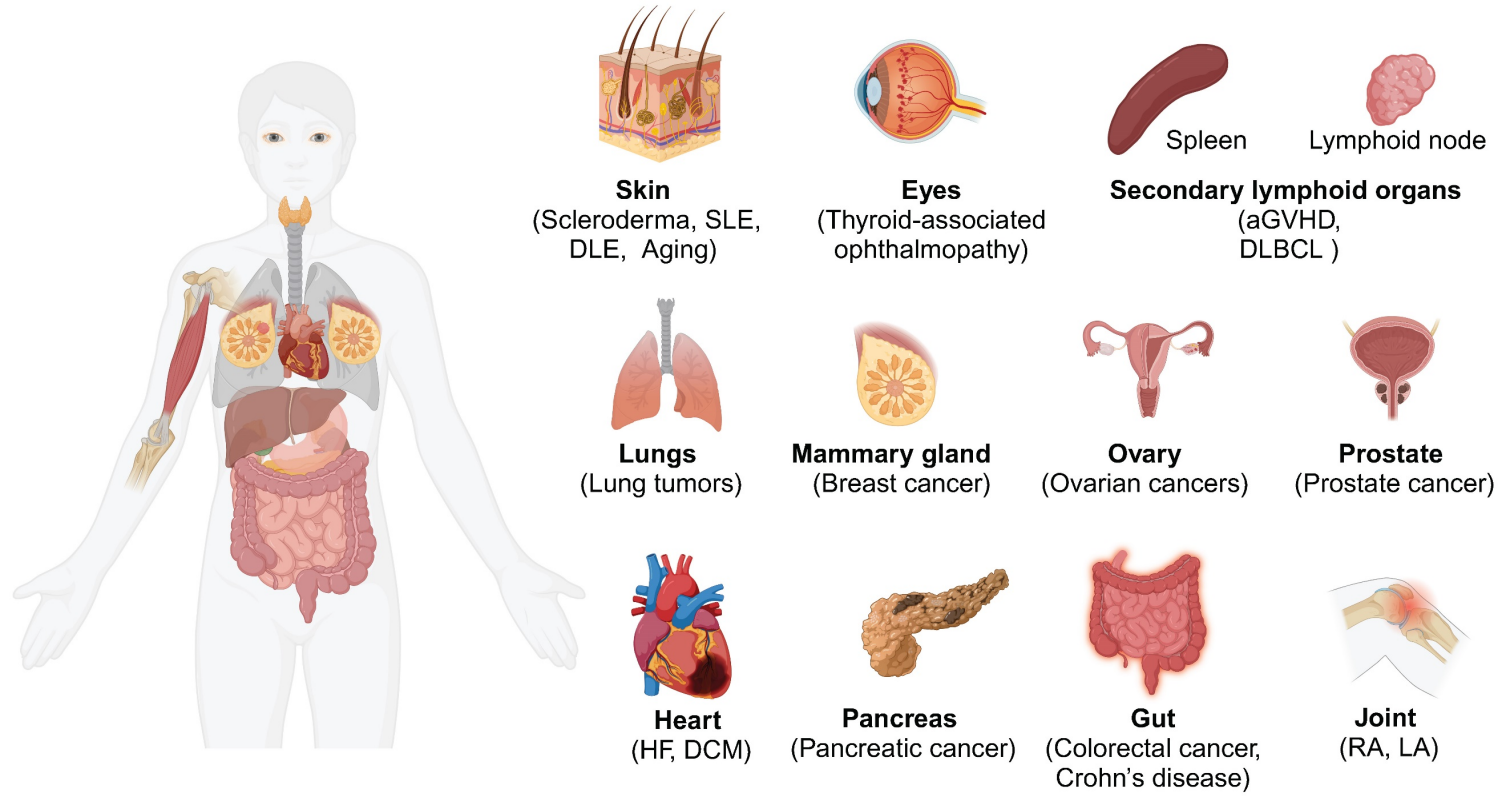
** Distribution of antigen-presenting fibroblasts.** The distribution of antigen-presenting fibroblasts in different human tissues and organs is characterized by the expression of CD74 and MHC-II molecules such as HLA-DRA and HLA-DRB1. Abbreviations: SLE, systemic lupus erythematosus; DLE, discoid lupus erythematosus; aGvHD: Acute graft versus host disease; DLBCL: Diffuse Large B-Cell Lymphoma; HF, heart failure; DCM, dilated cardiomyopathy; RA, Rheumatoid arthritis; LA, Lyme arthritis.

The presence of antigen-presenting fibroblasts has been confirmed across various human and murine tissues **(Figure 1)**, including the skin 18, heart 19, lung 9, gut 20, pancreas 21, breast 22, joint 23, lymph nodes 24, and others 25. Moreover, a recent integrative cross-disease analysis confirmed the widespread presence of CD74^+^ antigen-presenting fibroblasts in multiple organs and classified antigen-presenting fibroblasts as "shared" clusters 26. While these antigen-presenting fibroblasts are present in low numbers under normal physiological conditions (e.g., in the fat pads of the pancreas and breast), their frequency and expression of inflammatory genes such as IL32 and CXCR4 increase markedly in inflammatory or malignant conditions 22. This finding highlights the importance of considering fibroblasts within the broader immune landscape, particularly in disease settings where their antigen-presenting functions are amplified.

## 3. Origins and activation of antigen-presenting fibroblasts

**Figure 2:**
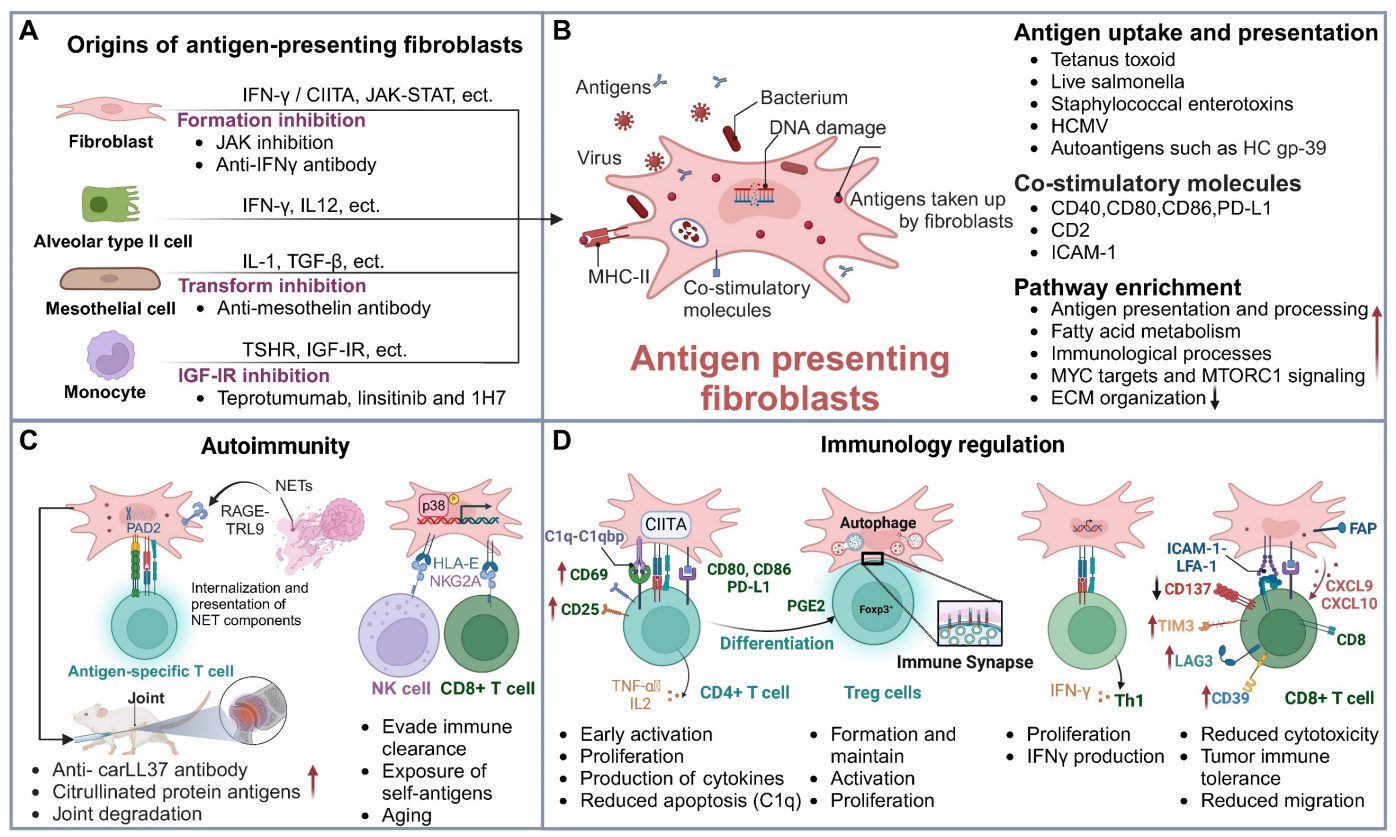
** Sources and functions of antigen-presenting fibroblasts.** (A) Origins of antigen-presenting fibroblasts and intervention strategies. This panel illustrates the various cellular origins of antigen-presenting fibroblasts, including fibroblasts derived from mesothelial cells, monocytes, and epithelial cells. Each origin is associated with distinct therapeutic interventions, such as JAK inhibitors, anti-mesothelin antibodies, and IGF-IR inhibitors, which target specific pathways to prevent their pathogenic activation. (B) Antigen uptake and presentation by antigen-presenting fibroblasts. This schematic shows the antigen-presenting fibroblasts' ability to phagocytose bacteria, viruses, and self-antigens. These cells present antigens via MHC-II molecules and express co-stimulatory molecules, such as CD80 and CD86, to modulate T cell responses. Their primary functions include antigen presentation and immune regulation. (C) Antigen-presenting fibroblasts in autoimmunity. NETs are internalized by FLS via the RAGE-TLR9 axis, leading to increased inflammatory responses. This process upregulates MHC-II expression, allowing FLS to present citrullinated NET-derived antigens to CD4^+^ T cells. FLS releases membrane-bound PAD2, promoting the citrullination of cartilage fragments. Elevated anti-carLL37 antibody levels and ACPA generation in joint inflammation result in synovial cartilage degradation and arthritis. Additionally, the expression of HLA-E on fibroblasts helps them evade immune clearance, further exposing self-antigens, and linking fibroblasts antigen presentation to autoimmune responses. (D) Immunoregulatory functions of antigen-presenting fibroblasts. Antigen-presenting fibroblasts influence the activation and function of various T cell subsets. They enhance T cell activation, promote differentiation of Tregs and Th1 cells, and affect the cytotoxic function of CD8^+^ T cells, thereby playing a key role in immune regulation across different disease contexts. Abbreviations: PAD2, peptidylarginine deiminase 2; FLS, fibroblast-like synoviocytes; carLL37, carbamylated LL37; NETs, neutrophil extracellular traps.

Fibroblasts exhibit considerable heterogeneity 27, 28, with IFN-γ being the critical driver for the formation of antigen-presenting fibroblasts. In addition to IFN-γ, other stimuli such as environmental factors and various signaling pathways can significantly modulate their antigen-presenting capabilities. Furthermore, a subset of antigen-presenting fibroblasts originates from the transformation of epithelial cells, mesothelial cells, and other cell types **(Figure 2A)**. Understanding the mechanisms that trigger their activation is crucial to unraveling their roles in immune responses and disease progression.

### 3.1 IFN-γ: A key driver of antigen-presenting fibroblasts

In several tissues such as the heart and synovium, IFN-γ induces the expression of MHC-II molecules in fibroblasts, highlighting its potential role in antigen presentation 19. Studies in IFN-γ or IFN-γ receptor-deficient mice have shown that MHC-II expression by fibroblasts is markedly reduced, further confirming the critical role of this cytokine 9. At the molecular level, IFN-γ activates key transcription factors such as CIITA through the JAK-STAT pathway, which is essential for driving MHC-II expression 29-31. Additionally, transcriptional regulators like CDK8 and CDK19 play an important role in the IFN-γ-mediated reprogramming of fibroblasts toward an antigen-presenting phenotype 32. While IFN-γ is a key factor, the precise regulatory mechanisms underlying MHC-II expression in fibroblasts remain to be fully understood.

In disease contexts, IFN-γ plays a significant role in shaping fibroblasts behavior. For instance, in RA, IFN-γ secreted by natural killer (NK) cells stimulates synovial fibroblasts, leading to the formation of HLA-DR^+^CD90^+^ fibroblasts, which contribute to joint inflammation 33. In the skin, IFN-γ produced by CD8^+^ T cells drives the upregulation of MHC-II and chemokines such as CXCL9 and CCL2, promoting immune cell recruitment 34. Additionally, specific risk single nucleotide polymorphisms (SNPs) associated with RA, such as rs6074022, have been shown to influence the expression of CD40 induced by IFN-γ, further emphasizing the complexity of IFN-γ-mediated fibroblasts activation in various tissues 35.

### 3.2 Additional stimulations and signaling pathways

In addition to IFN-γ signaling, various stimuli and environmental cues play critical roles in enhancing the antigen-presenting capabilities of fibroblasts. These factors include inflammatory signals, tissue injury, and metabolic reprogramming, which collectively influence fibroblasts activation and their ability to engage in antigen presentation. For instance, DNA damage has been shown to enhance the expression of MHC-I and co-stimulatory molecules on fibroblasts, boosting their antigen-presenting capacity 36. In the tumor microenvironment, antigen-presenting fibroblasts are closely associated with fatty acid metabolism 37. IL-12 also plays a crucial role in promoting the development of antigen-presenting fibroblasts, with a reduction in these cells observed in IL-12-deficient mice 9.

In certain conditions, fibroblasts with antigen-presenting capabilities also appear to originate from epithelial cells or monocytes. For instance, in pancreatic cancer, tumor-derived paracrine signals trigger the transformation of mesothelial cells into antigen-presenting fibroblasts, which exhibits upregulation of MHC-II molecules alongside mesothelial markers such as Msln, Upk3b, and Ezr 38. Similarly, IL-1 and TGF-β signaling pathways further enhance this transition, indicating that diverse pathways contribute to the formation of antigen-presenting fibroblasts in different disease contexts 21. In conditions like thyroid ophthalmopathy, a distinct subset of CD34^+^ fibroblasts displays enhanced MHC-II and co-stimulatory molecule expression, primarily originating from peripheral blood mononuclear cells (PBMCs) 39. Furthermore, in lung cancer, MHC-II^+^ fibroblasts are closely linked to alveolar epithelial cell gene signatures, suggesting that alveolar epithelial cells undergo EMT to become antigen-presenting fibroblasts.

## 4. Functional basis of antigen-presenting fibroblasts

Increasing evidence suggests that fibroblasts can assume the role of APCs under certain conditions. Their ability to present antigens relies on several key mechanisms, including antigen uptake and processing, as well as the expression of co-stimulatory molecules. Understanding these fundamental processes is crucial for revealing how fibroblasts contribute to immune regulation as important, non-professional APCs.

### 4.1 Antigen uptake and presentation

Antigen presentation begins with the recognition, phagocytosis, processing, and subsequent presentation of antigens. APCs possess sophisticated mechanisms for antigen uptake, including phagocytosis, receptor-mediated endocytosis, and macropinocytosis 40-42. Fibroblasts, while typically exhibiting limited antigen uptake under normal physiological conditions, can enhance their antigen acquisition abilities in pathological states, such as inflammation and cancer.

Infections caused by viruses and bacteria represent common sources of antigens 43, 44. Fibroblasts are capable of phagocytosing bacteria and processing antigens, and they can also be infected by viruses. For instance, dental pulp fibroblasts have been shown to produce C3b, facilitating the opsonization of bacteria and enhancing phagocytosis 45. During inflammation, apoptotic pathways and exocytosis receptors are upregulated in fibroblasts and immune cells, allowing dermal fibroblasts to effectively engulf apoptotic endothelial cells, a process important for tissue repair and fibrosis 46, 47. Moreover, fibroblasts can internalize polymeric nanocarriers through receptor-mediated endocytosis, influencing their functional responses and viruses can enter fibroblasts through pH- and kinetoprotein-dependent endocytosis 48, 49. Additionally, macropinocytosis facilitates the uptake of extracellular antigens and other substances. For example, glutamine deprivation drives fibroblasts to enhance macropinocytosis, supporting cancer cell adaptation 50. Notably, antigen-presenting fibroblasts can significantly increase their uptake capabilities upon stimulation, as demonstrated using ovalbumin (OVA) 21. This flexibility suggests that their antigen uptake functions are influenced by their activation state, microenvironmental cues, and cytokine stimulation. These characteristics underscore the crucial role of fibroblasts in immune responses, particularly in the processes of antigen uptake, processing, and presentation 33, 45, 51.

### 4.2 Expression of co-stimulatory molecules

The effectiveness of antigen presentation is contingent upon the duration and intensity of TCR-peptide-MHC interactions, as well as the balance of co-stimulatory and co-inhibitory signals. Professional APCs provide essential co-stimulatory signals, such as CD80, CD86, and CD40, necessary for complete T cell activation and proliferation 52-54. Under normal conditions, fibroblasts may lack these co-stimulatory molecules, which could limit their T cell activation capacity 55.

However, in pathological conditions, fibroblasts can upregulate co-stimulatory molecules, enhancing T cell activation. For example, in rheumatoid arthritis, synovial fibroblasts express CD80 and CD86, promoting the activation of autoreactive T cells and perpetuating inflammation 39. Furthermore, fibroblasts can express additional co-stimulatory factors, such as CD2, which is vital for T cell activation, particularly in inflammatory contexts 56. The expression of molecules like ICAM-1 on fibroblasts can further enhance T cell activation, as seen in psoriasis 57, 58. Additionally, fibroblasts can express co-stimulatory molecules like 4-1BBL, OX-40L, and CD70, which are associated with the activation and maintenance of memory T cells 59. While fibroblasts were traditionally thought to be less effective than professional APCs in antigen presentation, some studies have shown that their T cell-activating potential can be comparable to that of dendritic cells and B cells under certain conditions 20.

In summary, antigen-presenting fibroblasts are capable of both antigen uptake and presentation, as well as the expression of co-stimulatory molecules **(Figure 2B)**. While their functional capacity may vary under different conditions, both professional APCs and fibroblasts are essential in modulating immune responses. Understanding the mechanisms underlying their interaction is a key area of ongoing research.

## 5. Antigen-presenting fibroblasts in different diseases

Recent research has unveiled multifaceted functions of fibroblasts in immune responses, extending beyond their traditional roles in tissue structure to actively modulate immune reactions through antigen presentation. Their interactions with immune cells, such as NK cells and T cells 60, significantly impact the local immune microenvironment in cancer, autoimmune diseases, infections, and tissue injuries** (Figure 2C-D)**. Below, we discuss the specific functions of antigen-presenting fibroblasts in various disease contexts.

### 5.1 Cancer-associated antigen-presenting fibroblasts

In the tumor microenvironment, cancer-associated fibroblasts (CAFs) are key constituents of the stroma and contribute to tumor progression and immune evasion 38, 61. A subset of these fibroblasts, termed antigen-presenting cancer-associated fibroblasts (apCAFs), express MHC-II molecules and interact with T cells in an antigen-specific manner. In PDAC, mesothelial cells can transition into antigen-presenting fibroblasts, which induce T cell expression of early activation markers CD25 and CD69 in an OVA-specific manner, while other CAF subtypes cannot 21. In triple-negative breast cancer (TNBC) and pancreatic ductal adenocarcinoma (PDAC), apCAFs exhibit molecular signatures indicative of immunomodulatory functions 22. In colorectal cancer, apCAFs can elicit T cell reactivity against mismatched fibroblast cell lines loaded with mutated SLP, promoting the activation of CD4^+^ T cells, as evidenced by increased expression of CD69 and CD25. However, this interaction also reduces the cytotoxicity and activation of CD8^+^ T cells, suggesting a dual role of apCAFs in modulating the immune response.

Additionally, apCAFs promote the formation of Tregs in colonic tissues, dependent on MHC-II and prostaglandin E2 (PGE2) 62. In melanoma, α-SMA^+^ fibroblasts form an immunological synapse with Foxp3^+^ Tregs in the tumor microenvironment and instruct their activation and proliferation in an antigen-specific manner 63. This interaction highlights the significant role of fibroblasts in facilitating tumor immune evasion by inducing the formation of Tregs and suppressing effector T cell functions, thus potentially promoting tumor progression 64. Most importantly, the influence of a decrease in activating (CD137) and an increase in inhibitory (TIM3, LAG3, and CD39) checkpoint molecule expression on CD8^+^ T cells may lead to chemoresistance and decreased efficacy of chemotherapeutic drugs 20. This illustrates the complex interplay by which apCAFs regulate immune responses via antigen presentation.

While apCAFs primarily exist in malignancy, their exact roles in metastatic cancers are not fully understood. It remains unclear whether their presence enhances tumor immunity or correlates with prognosis 65-68. Despite their potential to promote immune suppression, CAFs can also enhance antitumor immunity by expressing molecules such as CD1d, which can activate NK cells and iNKT cells, leading to the destruction of tumor cells 69. At the same time, apCAFs can also prevent T cells from apoptosis through the action of C1q and C1qbp, thus inhibiting tumor formation and exerting an anti-tumor effect 9. This dual functionality suggests that the specific role of APFs depends on the cancer type and microenvironmental context.

### 5.2 Antigen-presenting fibroblasts in the skin

Dermal fibroblasts are essential for maintaining skin integrity and are primarily involved in synthesizing and renewing the extracellular matrix 70, 71. Aging skin typically shows a decline in immune surveillance, leading to increased exposure to self-antigens and potential activation of autoimmune responses 72, 73. In aged skin, the emergence of antigen-presenting fibroblasts is marked by a significant increase in HLA-II^+^ senescent fibroblasts. These cells are closely associated with heightened susceptibility to viral and bacterial infections, notably evidenced by the expression of HCMV RNA in aged dermal fibroblasts. Notably, these fibroblasts can present HCMV-gB to CD4^+^ T cells in an HLA-II-dependent manner, further activating T cells 74. Senescent fibroblasts expressing HLA-E can evade immune clearance by binding to the inhibitory ligand NKG2A, which is recognized by CD8^+^ T cells and NK cells. Concurrently, this interaction also modulates the functionality of CD8^+^ T cells and NK cells 60, 75.

Scleroderma is a unique autoimmune skin disease. It was previously believed that the abnormal activation and proliferation of myofibroblasts were key factors in its pathogenesis 76-78. When PBMCs are co-cultured with fibroblasts obtained from scleroderma patients, T cell expansion identical to that found in the skin of scleroderma patients and patient-derived PBMCs can be detected 79. This suggests the presence of a subset of fibroblasts in scleroderma skin capable of activating T cells. Recent studies have found elevated levels of antigen-presenting fibroblasts in localized scleroderma which highly express HLA-related genes (HLA-DQB1, -DPA1, -DRB1, -DRA) and inflammatory gene expression (CXCR4, CD74, IL32). Although this is a small subset of fibroblasts, cell-cell communication indicates an enrichment of ligand-receptor pairs and significant interactions with immune cells 18. Dermal HLA-DR-positive fibroblasts can present TT to autologous TT-specific monoclonal helper T cells 12. In lupus skin lesions, a group of antigen-presenting fibroblasts characterized by CD74 and HLA-DRB1 has also been identified, significantly enriched in pathways related to antigen presentation and cytokine production, which highlights the importance of these cells in autoimmune-related skin diseases 80.

### 5.3 Antigen-presenting fibroblasts in joints

In RA, synovial fibroblasts are primary inflammatory effector cells that present autoantigens to T cells, directly contributing to the autoimmune response 81, 82. They can present peptides from autoantigens such as HC gp-39 and human CII to antigen-specific MHC-restricted T cell hybridomas, functioning as antigen-presenting cells and directly interacting with infiltrating T cells *in vitro* 23. Synovial fibroblasts can up-regulate the activation marker CD69 in CD4^+^ T cells. When infected with Borrelia burgdorferi, MHC-II^+^ synovial fibroblasts are inducible antigen-presenting cells that can induce CD4^+^ T cell activation in an antigen- and CD40-dependent manner 17. Activated synovial fibroblasts can also present ECM-derived Lyme autoantigens, implicating them in amplifying tissue-localized autoimmunity in LA 17.

Proteins from neutrophil extracellular traps (NETs) constitute a significant portion of the autoantigen pool in RA 83. Synovial fibroblasts can also internalize NETs, presenting citrullinated antigens to T cells, thus contributing to autoantibody production and joint degradation. NET-derived proteins, such as carLL37, are processed and presented by synovial fibroblasts, eliciting autoantibody responses that further promote inflammation and damage in RA. This antigen-presenting function of fibroblasts in the synovium links them closely to autoimmune responses and disease progression in RA 82, 84, 85.

### 5.4 Antigen-presenting fibroblasts in other tissues and organs

In cardiac tissue, fibroblasts play a crucial role in tissue repair after myocardial infarction 86, 87. Recent studies have further confirmed the presence of a subset of cardiac fibroblasts characterized by the expression of MHC-II molecules 19. These cells are significantly expanded in heart failure and dilated cardiomyopathy and can effectively present antigens, promoting T cell activation 88. Notably, the specific deletion of MHC-II in fibroblasts can effectively alleviate cardiac remodeling and dysfunction induced by transverse aortic constriction (TAC). This is a significant breakthrough as it not only identifies this subset from the single-cell sequencing perspective but also validates the role of antigen-presenting fibroblasts in the development of cardiac diseases through *in vivo* experiments.

Fibroblasts also contribute to immune regulation in the lymph nodes, where fibroblastic reticular cells (FRCs) can present antigens to CD8^+^ T cells and inhibit their cytotoxic activity, promoting immune evasion in conditions like diffuse large B cell lymphoma (DLBCL). Intrinsic MHC-II expression in lymph node stromal cells promotes the transformation of MHC-I-restricted CD8^+^ T cell lineage into regulatory CD4^+^ T cells 89. The activated LNSC acquires enhanced antigenic presentation and acts as an external brake system for CD4^+^ T cell response. FRCs located in the T cell zone of LN ectopically express and directly present model PTA to naive T cells, thereby inducing their proliferation 90, 91.

In addition to their direct antigen-presenting functions, HLA-DR expressed on fibroblasts can also act as a receptor molecule that transmits signals to fibroblasts based on DR-peptide-TCR interactions, resulting in the secretion of multiple cytokines 92. This communication is often mediated through the secretion of cytokines and chemokines, which can significantly alter the immune landscape of a given tissue or organ. For example, cytokines produced by macrophages, such as IL-1β, IFN-γ, and TNF, can profoundly affect fibroblasts gene expression, leading to the production of CXCL and CCL ligands. These chemokines, in turn, can recruit and activate various immune cells, thereby shaping the immune response in a tissue-specific manner. Such interactions are especially relevant in chronic inflammatory conditions, where fibroblasts can either amplify or attenuate ongoing immune responses.

## 6. Diagnosis and therapeutic implications

The functional diversity of antigen-presenting fibroblasts highlights their significant roles in modulating immune responses across a spectrum of diseases **(Table 1)**. This makes them attractive targets for therapeutic interventions aimed at either enhancing their immunostimulatory functions in cancer or inhibiting their pro-inflammatory roles in autoimmune diseases. Below, we explore their diagnostic potential and therapeutic strategies aimed at modulating their functions.

### 6.1 Diagnostic potential

The expression of MHC-II molecules has emerged as a robust prognostic marker for immune responses and a predictor of outcomes in patients undergoing immune checkpoint inhibitor therapy 98. In prostate cancer, an increased presence of fibroblasts involved in antigen presentation and processing has been observed, with their antigen-presentation gene expression serving as a predictive marker of disease risk 65. These antigen-presenting fibroblasts exhibit significant interaction with T cells and are strongly correlated with immune cell infiltration, highlighting their potential as biomarkers for disease progression in cancer and autoimmune disorders 65.

Moreover, imaging techniques targeting fibroblast activation protein (FAP) have shown significant promise in tumor diagnosis, particularly in metastatic cancers such as prostate cancer 99. FAP is a marker that is frequently upregulated on activated fibroblasts, highly expressed in various malignancies, including cancer, fibrotic diseases, and inflammatory conditions 100, 101. The use of selective tracers like Gallium 68 (^68^Ga)-labeled fibroblast activation protein inhibitor (FAPI) in PET imaging has demonstrated excellent diagnostic accuracy by visualizing activated fibroblasts 102, 103. Integrating FAPI-PET imaging with MHC-II-targeted imaging techniques (e.g., MHC-II probes and molecular imaging) may offer a non-invasive method to assess antigen-presenting fibroblasts in diseases such as tumors, fibrosis, and chronic inflammation 104.

### 6.2 Therapeutic implications

**Figure 3:**
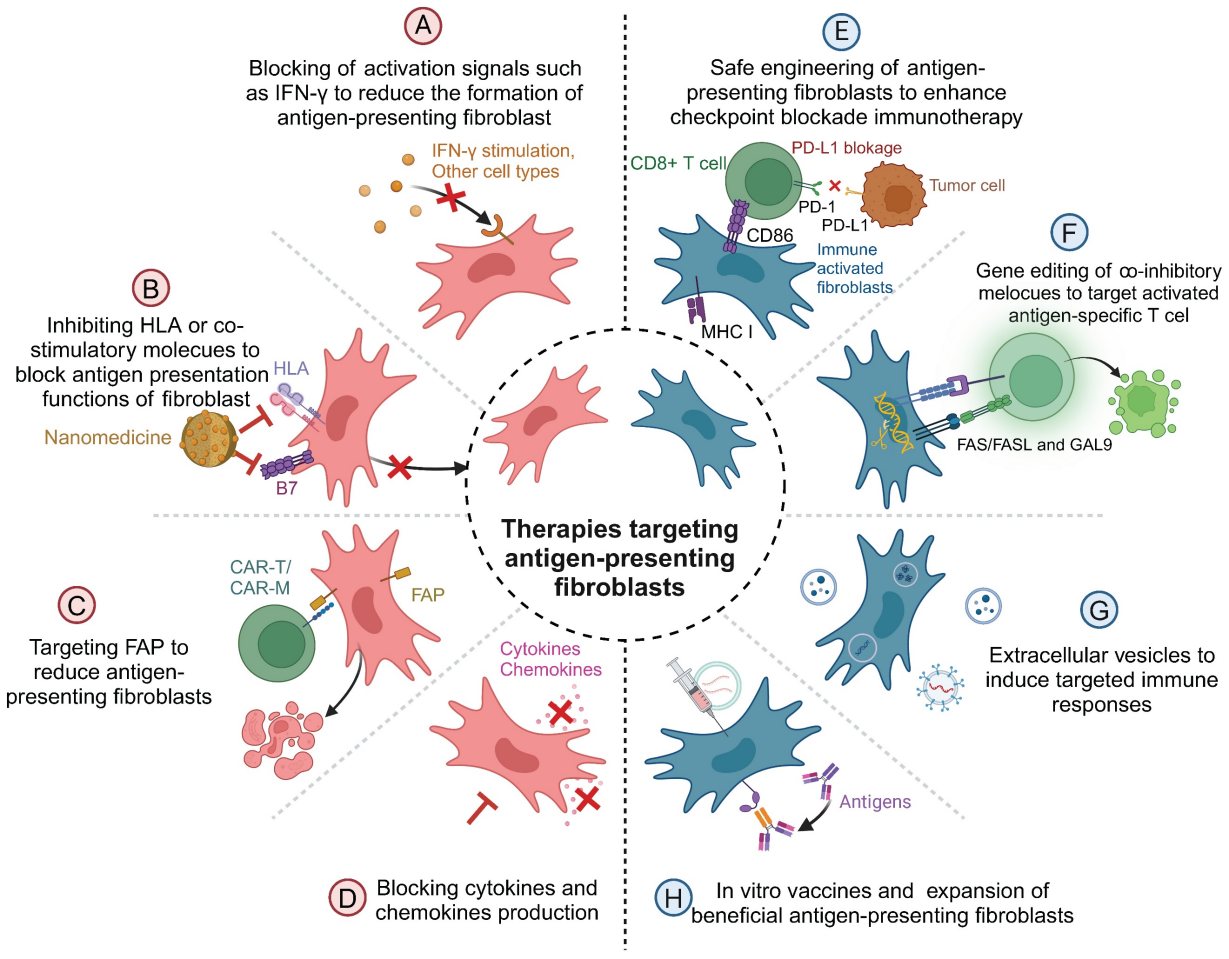
Therapeutic interventions on antigen-presenting fibroblasts.

Antigen-presenting fibroblasts play dual roles in diseases, serving as both mediators of immune activation and regulators of immune responses. This dual functionality renders them promising therapeutic targets for a range of diseases, including autoimmune disorders and cancers. Therapeutic strategies aimed at modulating antigen-presenting fibroblasts focus on inhibiting their formation, disrupting their antigen-presentation functions, or selectively eliminating them, depending on the disease context **(Figure 3)**.

One approach to targeting antigen-presenting fibroblasts is to inhibit their differentiation and formation. For instance, JAK inhibitors have been primarily designed to suppress IFN-γ-induced activation of fibroblasts, thereby reducing their antigen-presenting capacity and mitigating autoreactive T cell activation in autoimmune diseases like RA 33. Similarly, anti-mesothelin antibodies have been employed to block the transformation of mesothelial cells into antigen-presenting fibroblasts. This strategy has been demonstrated to enhance antitumor immunity, particularly in cancers such as pancreatic cancer 21. Additionally, IGF-IR inhibitors reduce the generation of monocyte-derived antigen-presenting fibroblasts, effectively dampening pathological immune responses 39.

Beyond inhibiting their formation, therapies that disrupt the functional components of antigen-presenting fibroblasts provide another promising avenue. Strategies such as blocking MHC-II molecules using specific antibodies or nanotechnology-based systems have shown the potential to impair the antigen-presentation capabilities of these cells 105-107. Gene-editing techniques, including those that inhibit autophagy or disrupt MHC-II expression, represent additional tools for reducing the immunopathological roles of antigen-presenting fibroblasts 108. Such interventions offer therapeutic benefits in both autoimmune diseases and cancers, where aberrant antigen presentation drives disease progression.

Selective elimination of antigen-presenting fibroblasts has also emerged as an innovative therapeutic strategy, particularly in oncology. CAR-T cells engineered to target FAP, a marker widely expressed on pathogenic fibroblasts, have demonstrated significant potential in preclinical studies 109, 110. Advanced CAR-T constructs, such as UniCAR modules and CAR-TEAM designs, enhance specificity while reducing the risk of off-target effects 111, 112. Furthermore, CAR-macrophages, which possess superior tissue infiltration capabilities compared to CAR-T cells, have been employed to target fibroblasts in dense tumor microenvironments, broadening the scope of therapeutic options 100.

In addition to these direct interventions, the mediators secreted by antigen-presenting fibroblasts also present therapeutic targets. Neutralizing pro-inflammatory cytokines or blocking downstream signaling pathways has been shown to mitigate the pathological effects of antigen-presenting fibroblasts. Conversely, harnessing fibroblast-derived exosomes carrying therapeutic molecules, such as anti-inflammatory genes, offers novel opportunities for immunomodulation. For instance, tumor-associated fibroblast-derived exosomes carrying lncRNAs can influence immune evasion by downregulating HLA expression in pancreatic cancer, providing a potential therapeutic tool for both autoimmune diseases and cancers 113, 114.

Reprogramming antigen-presenting fibroblasts to enhance their immune-stimulatory properties represents another exciting frontier. Advances in genetic engineering have enabled the modification of fibroblasts to overexpress co-stimulatory molecules like CD86, thereby amplifying CD8^+^ T cell responses and improving the efficacy of immune checkpoint blockade therapies 10. *In vivo*, vaccination strategies that deliver antigens directly to antigen-presenting fibroblasts or enhance their antigen-presentation capacity have been shown to promote local immune responses and systemic immune protection 115. Additionally, inducing regulatory phenotypes in fibroblasts by stimulating the expression of molecules such as Gal-9 or Fas/FasL offers a novel approach for suppressing aberrant immune responses in autoimmune diseases 116.

The therapeutic potential of targeting antigen-presenting fibroblasts underscores the need for context-specific strategies. In cancers, the focus is on suppressing the immunosuppressive functions of antigen-presenting fibroblasts to enhance cytotoxic T cell activity, thereby promoting antitumor immunity 117-121. Conversely, in autoimmune diseases, therapies aim to inhibit autoreactive T cell responses and restore immune tolerance 122-125. These contrasting goals highlight the versatility of antigen-presenting fibroblasts-targeted therapies in addressing diverse pathological conditions.

As our understanding of antigen-presenting fibroblasts continues to evolve, advances in diagnostic tools to assess their activity in patient tissues will play a crucial role in guiding personalized treatment strategies. By precisely manipulating the biological functions of antigen-presenting fibroblasts, these cells can be transformed from disease mediators into therapeutic allies. Such progress holds significant promise for innovative treatments in cancer, autoimmune diseases, and beyond.

## 7. Conclusion

Fibroblasts have evolved from being regarded solely as structural components to key players in immune regulation, with their antigen-presenting capabilities redefining their role in pathology. Their dual functions underscore their significance in disease progression and therapeutic intervention. Understanding the molecular mechanisms and contextual cues that govern their antigen-presenting functions is essential for unlocking their full potential. This knowledge will not only deepen our comprehension of fibroblasts biology but also guide the development of innovative diagnostic tools and therapies, positioning fibroblasts at the forefront of immune modulation and disease management.

## Figures and Tables

**Table 1:** Antigen-Presenting fibroblasts in various tissues and organs

Species	Diseases	Tissue	Name	Markers	Functions	References
Human	Ovarian cancers	Ovary	apCAFs	HLA-DRA, HLA-DRB	ApCAFs were found in various metastatic tumor foci.	66
Mouse	PDAC	Pancreas	apCAFs	Histocompatibility 2, class II antigen A, alpha (H2-Aa) and beta 1 (H2-Ab1), Serum Amyloid A3, Cd74, Slpi, stata1	Antigen presentation and processing; Fatty acid metabolism; MYC targets and MTORC1 signaling.	8
Human	PDAC	Pancreas	apCAFs	HLA-DRA, HLA-DPA1 and HLA-DQA1, SLPI, CD74, XBP1	ApCAFs isolated from the same orthotopic tumors demonstrated the capacity to induce CD25 and CD69 in co-culture 2d T cells in an OVA-specific manner.	8
Human,Mouse	Pancreatic cancer	Pancreas	apCAFs	MHC-II, Cd74, H2-Aa, H2-Ab1, H2-Dma, H2-Dmb1, H2-Eb1	Expanded specifically in all late-stage GEMMs; ApCAFs induce naive CD4^+^ T cells into regulatory T cells, induce Tregs formation and expansion; Induced CD4^+^ T cell expression of early activation markers, CD25 and CD69.	21
Mouse	Pancreatic cancer	Pancreas	apCAFs	Cd74, H2-Ab1, Saa3	ApCAFs clustered with mesothelial cells from normal pancreas.	93
Human	Colorectal cancer	Gut	apCAFs	HLA A3^-^B7^-^, A3^+^B7- or A3^-^B7^+^	T cell activation, induced production of TNFα and IL-2; Impairs CD8^+^ T cell effector function Result in a decrease in activating (CD137) and increase in inhibitory (TIM3, LAG3 and CD39) checkpoint molecule.	20
Human	\	Gut	Subepithelial myofibroblasts	αSMA^+^, CD90^+^, MHC-II, CD80, CD86	Able to stimulate allogeneic CD4^+^ T cell proliferation.	94
Human	Staphylococcal enterotoxigenic disease	Gut	Subepithelial intestinal myofibroblasts	MHC-II	IMFs bind staphylococcal enterotoxins in an MHC-II-dependent fashion *in vitro*.	95
Human	Salmonella typhimurium infection	Gut	Intestinal stromal cells	CD90^+^ HLA-DR	Rapid internalization and sensing of live Salmonella; Capacity for phagocytosis and antigen processing.	96
Human	\	Gut	Colonic myofibroblasts/fibroblasts	CD90^+^, αSMA^+^ MHC-II	Contribute to the maintenance of FoxP3^+^ phenotype of the nTregs; Induce generation of iTregs from naïve CD4^+^ T cells (MHC-II- and PGE2- dependent).	62
Mouse	Breast Cancer (Mammary Tumors)	Breast	apCAFs	H2-Aa, H2-Ab1, H2-Eb, Cd74, Krt7, Krt8 and Krt18 and Fsp1 (S100a4)	Immune modulatory role in the tumor microenvironment.	22
Human, Mouse	Lung tumors	Lungs	apCAFs	MHC-II and FAP, Lin^-^	ApCAFs activate adjacent CD4 T cells.	9
Human	Primary NSCLC with metastasis	Lungs	apCAFs	HLA-DRA, HLA-DPB1, HLA-DPA1, SP11, RUNX3, MAF	ApCAF may play a role in bone metastasis by activating signalling pathways associated with cancer stemness, such as SPP1-CD44 and SPP1-PTGER4.	67
Human	NTHi infection	Lungs	Fibroblasts	CD45^-^EpCAM^-^CD90^+^HLA-DR^+^	Able to present Ag and activate bacteria-specific autologous Th cells when preconditioned with IFN-γ.	59
Human	Inflamed joint tissues	Joint	FLS	MHC-II	Present peptides from the autoantigens HC gp-39 and human CII to antigen-specific MHC-restricted T cell hybridomas.	23
Human	RA	Joint	FLS	HLA-DR^+^CD90^+^, HLA-DRB1, HLA-ABC, CD54	Up-regulated the activation marker CD69 in CD4^+^ T cells.	33
Human	LA	Joint	FLS	HLA-DR	Induce CD4^+^ T cell activation in an antigen- and CD40-dependent manner; Present ECM-derived Lyme autoantigens, implicating FLS in amplifying tissue-localized autoimmunity in LA.	17
Human	Infection	Skin	HLA-DR^+^ fibroblasts	HLA-DR	Present tetanus toxoid (TT) to autologous TT-specific monoclonal helper T cells; Induce significant proliferation of cloned T cells.	12
Human	Localized Scleroderma	Skin	Cluster 11 (CD74/ DUSP2)	DUSP2, CD74, HLA-B, HLA-C, HLA-DRB1, HLADRA, CXCR4, CD74, IL32	Unique fibroblast populations in LS compared to controls; Potential roles for fibroblasts through cell-cell communication and trajectory software, especially macrophage interaction.	18
Human	SLE	Skin	HLA^+^ fib	HLA-DRA, HLA-DRB1, CD74	Function as nonclassical antigen-presenting cells.	80
Human	Aging	Skin	HLA-II ^+^ fib	HLA-II, HCMV-gB	Act as inducible antigen-presenting cells that can activate CD4 CTL from the human skin and participate in immune regulation.	74
Mouse	Melanoma	Skin	α-SMA^+^ CAFs	Cd74, H2-Aa, H2-DMa and H2DMb1	Form an immunological synapse with Foxp3^+^ Tregs in the tumor microenvironment and instruct their activation and proliferation in an antigen-specific manner.	63
Human, Mouse	DLBCL	Lymph node	DLBCL activated FRCs	HLA genes, B2m and Cd74	Inhibit CD8^+^ TIL cytotoxicity in an antigen-specific manner Reduced ability to promote T lymphocyte migration.	64
Mouse	HF	Heart	Cardiac fibroblasts	MHC-II	Induce naïve CD4^+^ T cell proliferation; Promote antigen-specific Th1 cell activation Be central to cardiac remodelling and dysfunction in response to TAC.	19
Mouse	aGvHD	Secondary lymphoid organs	CCL19^+^ FRCs	Cd74, Tap2, H2-Aa, H2-Eb1, H2-Eb2, and H2-Ab1, CCL19	Promotes the expansion of antigen-specific Tregs and controls T cell alloreactivity in the effector phase of GvHD.	97

## Abbreviations

MHC-II: major histocompatibility complex class II
APCs: antigen-presenting cells
SLE: systemic lupus erythematosus
DLE: discoid lupus erythematosus
HF: heart failure
DCM: dilated cardiomyopathy
RA: rheumatoid arthritis
NK: natural killer
Tregs: regulatory T cells
SNPs: single nucleotide polymorphisms
PBMCs: peripheral blood mononuclear cells
EMT: epithelial-mesenchymal transition
FRCs: fibroblastic reticular cells
OVA: ovalbumin
PAD2: peptidylarginine deiminase 2
FLS: fibroblast-like synoviocytes
CarLL37: carbamylated LL37
NETs: neutrophil extracellular traps
LA: lyme arthritis
GvHD: graft versus host disease
aGvHD: acute graft versus host disease
DLBCL: diffuse large B-cell lymphoma
CAFs: cancer-associated fibroblasts
ApCAFs: antigen-presenting cancer-associated fibroblasts
TAC: transverse aortic constriction
TNBC: triple-negative breast cancer
PDAC: pancreatic ductal adenocarcinoma
TT: tetanus toxoid
FAP: fibroblast activation protein
FAPI: fibroblast activation protein inhibitor
HCMV: human cytomegalovirus
